# Long term stability of adsorption of Der s1 allergen onto alum at different phosphate ion concentrations

**DOI:** 10.1186/1939-4551-8-S1-A126

**Published:** 2015-04-08

**Authors:** Roxana Samalea Díaz, Wendy Ramírez González, Deibbys Martinez García, Alain Morejón, Yunia Oliva Diaz, Damaris Torralba Averoff, Arelis Más Quintero, Maytee Mateo, Alexis Labrada, Raysa Cruz

**Affiliations:** 1 National Center of Bioproducts, Cuba

## Background

Allergen adsorption onto alum is relevant for safety and immunogenicity of alum-adjuvanted allergen vaccines, used for subcutaneous immunotherapy. Adsorption of the Group 1 Mite allergens can be impaired by phosphate buffers. On the other hand, diminishing or abolishing buffer salts can affect long term stability. Alum content can also influence immunogenicity and adsorption, as well as, stability.

## Aim

To evaluate the stability of formulation variants of a *Dermatophagoides siboney* adjuvanted vaccine, using different content of phosphate salts and alum.

## Methods

There were prepared 3 batches of each of four formulation variants of the allergen vaccine PROLINEM-DS at pilot scale, complying with GMP. The vaccine contains an allergen fraction of *D.siboney* extract, alum and proteoliposome of *Neisseria meningitidis*as a TLR4 ligand. Variants consisted of a gradual reduction of phosphate ion and alum (from 1 to 2mg/mL). Adsorption of Der s1 was assessed by Mab-ELISA. Residual non-adsorbed total allergenic activity was measured by IgE inhibition ELISA. A real time stability study was performed at 4°C testing at 0, 3, 6, 9, 12 during 18 months, according to ICH guidelines.

## Results

The highest value of Der s1 adsorption was 98,7% achieved for the variant without phosphate, whereas the variant with full PBS buffer showed only 85,2%. After 18 months of storage at 4°C, the adsorption values remained high at 98,8% for the variants with reduced phosphate content, and even raised up to 94,8% for the variant with the highest phosphate content. Similar behaviour was noted for the non-adsorbed allergenic activity, in line with the major role of Der s1 allergen as IgE binding component within the allergen extract; pH values did not show changes overtime even in absence of phosphate buffer. Other quality control tests showed results according to release specifications. Linear regression analysis for Der s1 adsorption and residual allergenic activity confirmed a significant trend (p<0.05) towards an increase of adsorption values overtime in the variants with phosphate content. Alum content did not show a significant influence on adsorption and remained stable, as well, during 18 months. Batch to batch consistency of all formulation variant was demonstrated statistically.

## Conclusions

Long term stability of allergen adsorption onto alum was demonstrated for a wide range of phosphate buffer as well as alum content variation, which provide a basis for the concept of “design space”, thus, assuring the safety and immunogenicity of the novel Prolinem-DS allergen vaccine.

